# Obtaining Genome Sequences of Mutualistic Bacteria in Single *Microcystis* Colonies

**DOI:** 10.3390/ijms20205047

**Published:** 2019-10-11

**Authors:** Jing Tu, Liang Chen, Shen Gao, Junyi Zhang, Changwei Bi, Yuhan Tao, Na Lu, Zuhong Lu

**Affiliations:** 1 State Key Lab of Bioelectronics, School of Biological Science and Medical Engineering, Southeast University, Nanjing 210096, China; tongust@163.com (L.C.); gaoshen12345gg@hotmail.com (S.G.); blocksharon@163.com (J.Z.); bichwei@163.com (C.B.); 220174561@seu.edu.cn (Y.T.); nlu@seu.edu.cn (N.L.); 2 Wuxi Environmental Monitoring Center, Wuxi 210096, China

**Keywords:** cyanobacterial blooms, *Microcystis*, metagenomics, single *Microcystis* colony, mutualistic bacteria

## Abstract

Cells of *Microcystis* are associated with heterotrophic bacteria and organized in colonies in natural environment, which are basic elements in the mass occurrence of cyanobacterial species. Analyzing these colonies by using metagenomics is helpful to understand species composition and relationship. Meanwhile, the difference in population abundance among *Microcystis* colonies could be used to recover genome bins from metagenome assemblies. Herein, we designed a pipeline to obtain high-quality genomes of mutualistic bacteria from single natural *Microcystis* colonies. Single colonies were lysed, and then amplified by using multiple displacement amplification to overcome the DNA quantity limit. A two-step assembly was performed after sequencing and scaffolds were grouped into putative bins based on their differential-coverage among species. We analyzed six natural colonies of three prevailing *Microcystis* species from Lake Taihu. Clustering results proved that colonies of the same species were similar in the microbial community composition. Eight putative population genome bins with wide bacterial diversity and different GC content were identified based on coverage difference among colonies. At the phylum level, proteobacteria was the most abundant besides cyanobacteria. Six of the population bins were further refined into nearly complete genomes (completeness > 90%).

## 1. Introduction

Cyanobacterial blooms occur as a result of massive proliferation of cyanobacteria [1], and have become a worldwide environmental problem [2]. Among several cyanobacterial genera, the unicellular, worldwide distributed cyanobacterium *Microcystis* is the most representative genus of bloom-forming cyanobacteria [3,4].

In natural environment, *Microcystis* cells are associated with heterotrophic bacteria and organized in colonies which vary in sizes and shapes [5]. The rise and accumulation of large *Microcystis* colonies is the immediate cause of abrupt occurrence of *Microcystis* blooms [6]. Lab culture experiments support that the natural heterotrophic bacterial community have a role in the development of *Microcystis* blooms in natural water [7]. To improve our understanding of the relationship within *Microcystis* colonies, genomes of representative heterotrophic bacteria are desired to be obtained. Systematically sequencing genomes from pure cultures is an effective method to obtain bacteria genomes. However, it is challenging to disassociate and purify these heterotrophic bacteria from *Microcystis* colonies and only a small portion of them is representable by cultivated organisms [8]. By directly extracting bulk DNA from environmental samples and sequencing, metagenomics has overcome the disassociation and cultivation obstacles [9]. High-quality genomes of dominant populations have been assembled from relatively low-complexity communities using metagenomics [10,11]. Individual genomes can be recovered by grouping sequences of particular individuals from a background of community sequencing data [12]. The applications of metagenomics in cyanobacterial blooms [2,13,14,15] have revealed useful information on the microbial communities. However, the genomes of individual members are still not well assembled because the microbial communities of *Microcystis* blooms are high complex microorganism mixtures in natural freshwater. These mixtures contain different types of *Microcystis* colonies and some other planktonic microbial communities.

As the basic element in the mass occurrence of cyanobacterial species, single *Microcystis* colony is less complex in species composition, is an ideal sample for metagenomic studies, and has been used in microcystin gene analysis [16,17] or genetic study [18]. *Microcystis* colonies differ in morphological characteristics and can be divided into different morphospecies [19]. Each type of *Microcystis* colony is diverse in microbial community and has different metabolic pathways among the members. Many researches attempted to understand the morphological variation among *Microcystis* in several directions [20,21,22,23,24,25]. However, because the DNA quantity is quite limited in a single *Microcystis* colony, it is difficult to perform efficient metagenome analysis. This bottleneck can be bypassed by culturing individual type of *Microcystis* colonies in lab. Xie et al. cultured *Microcystis wesenbergii* T100 in lab for bulk DNA extraction and downstream analysis, and the genomes of eight individual mutualistic bacteria were reconstructed from metagenomic assemblies [26]. However, some colonial *Microcystis* stains lose their mucilage and convert into individual cells gradually after some generations in lab culture [23]. The lab-maintained colonies are less complex and their microorganic compositions are unlike the ones in nature [7,26]. Single natural *Microcystis* colonies are more suitable for metagenomic studies to obtain the genome sequences of associated bacteria from the bacterial communities in cyanobacterial blooms. Meanwhile, the difference in population abundance among *Microcystis* colonies can be used to recover genome bins from metagenome assemblies.

Herein, we designed a pipeline to obtain genome sequences of mutualistic bacteria in single *Microcystis* colonies, which lysed single colonies and amplified the metagenomes before library construction, and grouped sequences of metagenome based on their differential-coverage among three prevailing species of *Microcystis* colonies. Six related metagenome sequencing libraries were generated by disassociating single *Microcystis* colonies from a mixed sample, following by multiple displacement amplification (MDA) to overcome the DNA quantity limit. The assembled metagenome scaffolds were grouped in a three-dimension space based on sequencing depth. We obtained eight population genome bins of the cohabitation bacteria which differed in abundance among *M. aeruginosa*, *M. wesenbergii* and *M. panniformis* colonies, and six of them were further refined into high-quality genomes.

## 2. Results

### 2.1. Binning of Single *Microcystis* Colony Metagenomes

**Figure 1:**
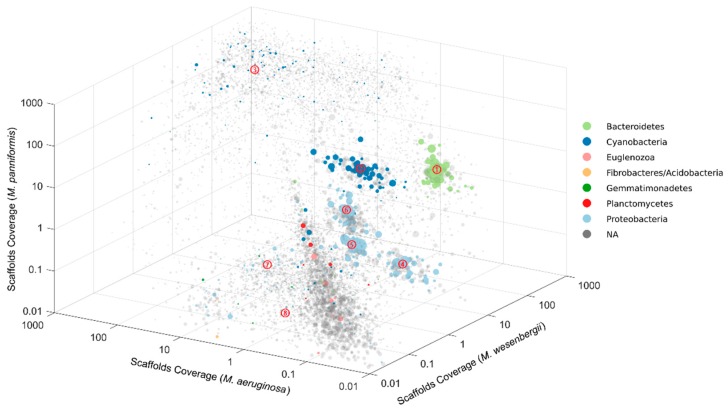
Sequence 3D coverage binning of metagenome scaffolds from the single *Microcystis* colonies. Plots represent scaffolds, the size indicates the length of scaffolds and the color shows the phylum-level taxonomic affiliation. Gray spots are scaffolds which cannot be assigned to the essential genes of the 8 most abundant phyla. Clusters of same colored spots represent potential genome bins, and are indicted by numbers in the center (Table 2). Spots in similar coverage pattern, such as around diagonal, were extracted and further binned based on the differences in nucleotide composition.

We deeply sequenced DNA from single *Microcystis* colonies of three species (*M. aeruginosa*, *M. wesenbergii* and *M. panniformis*) collected from Lake Taihu, the third largest freshwater lake in China, which suffers annual *Microcystis* blooms. A total of 87 Gbp of paired-end sequencing data were produced and were assembled into a total of 105 Mbp of scaffolds ranging in size from 1 Kbp to 198 Kbp (Table S1). Due to the abundance difference among species, populations were differentially represented in each data set. We firstly binned the scaffolds into population genomes by plotting all scaffolds in three-dimension (3D) based on their coverage in different *Microcystis* colonies (Figure 1 and Figure S2). Some subsets of spots were further binned based on the differences in nucleotide composition. In total, eight putative population genome bins with wide bacterial diversity and big range of GC content were identified, and six of them were further refined into high-quality genomes.

### 2.2. Pipeline of Obtaining Genomes in Single *Microcystis* Colonies

**Figure 2:**
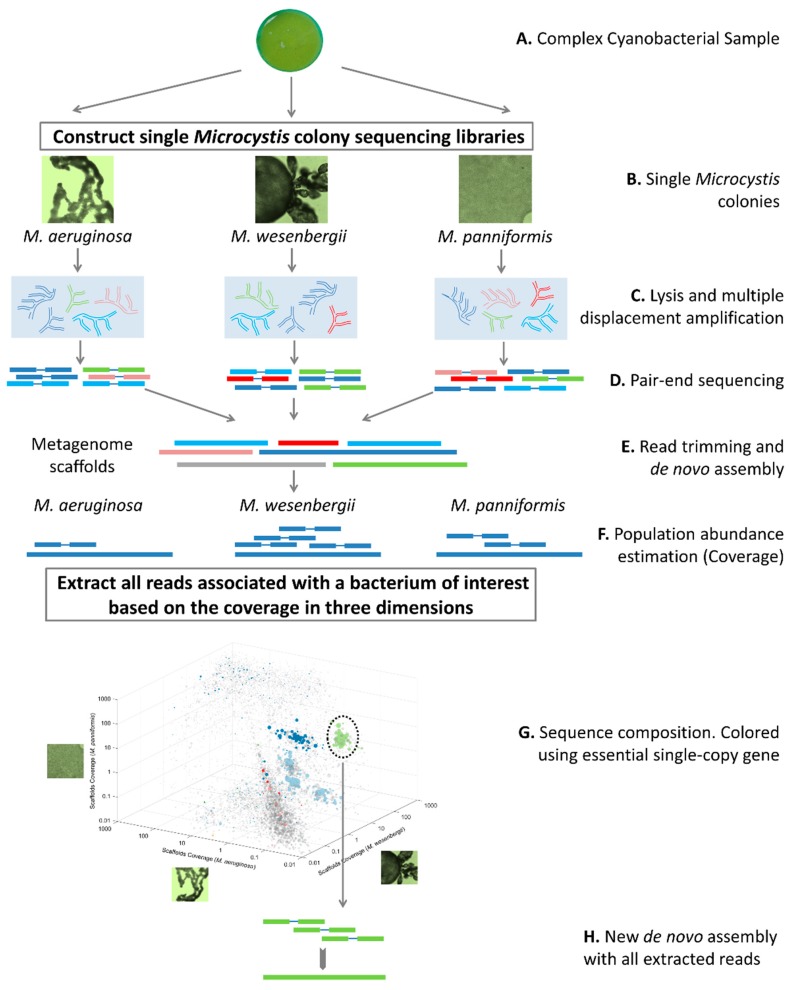
Overview of the pipeline to obtain population genomes from single *Microcystis* colony metagenomes. A. The complex cyanobacterial sample was collected. B. Colonies of three dominated *Microcystis* species (*M. aeruginosa*, *M. wesenbergii* and *M. panniformis*) were separated and classified based on their species features. C. Colonies were lysed and amplified by using MDA. D. Paired-end sequencing libraries were constructed and the metagenomes were sequenced separately. E. All data of metagenomes were used unitedly in de novo assembly after read trimming. F. The population abundance of each assembled scaffold was estimated based on its coverage in each metagenome. G. The binning of the scaffolds was facilitated by plotting coverages against each other. The colored spots are scaffolds which can be assigned to the essential genes of the 8 most abundant phyla. H. The subsets of scaffolds associated with the grouped bins were extracted. The paired-end reads which at least one end mapped to the scaffolds were extracted and used for a new de novo assembly.

The overall pipeline of obtaining genomes in single *Microcystis* colonies is illustrated in Figure 2. Briefly, colonies of *Microcystis* were separated from a cyanobacterial bloom sample collected from Lake Taihu, and were classified under light microscope based their species features. *Microcystis* colonies which were classified as *M. aeruginosa*, *M. wesenbergii* and *M. panniformis* were selected for metagenome analysis. These three species are dominated species in Lake Taihu. Two parallel colonies of each species were used for following analysis. Due to limit of DNA quantity in a single *Microcystis* colony, MDA, which is superior in genome coverage, amplification accuracy and efficiency [27,28], was used to amplify the lysed single colonies. Sequencing libraries were constructed using the amplified nucleotides and the six single colonies were sequenced separately. All the paired-end sequence data of the six samples were used unitedly in de novo assembly. After the evaluating parallel colonies, reads from the first one of two parallel colonies of each species were used for mapping to assembled scaffolds, and the mapping results showed that populations were differentially represented due to their diverse abundance in specific species. The binning of the scaffolds was firstly facilitated by plotting the three coverage against each other in the 3D space. Some putative genome bins were also purified based on their GC content and using principal component analysis of tetranucleotide frequencies. After that, all the sub-network of scaffolds associated with the grouped bins were extracted. To improve the assembly, all relevant reads of a genome bin were extracted for reassembly, including the paired-end reads which both ends mapped to the scaffolds and those which only one end mapped to the scaffolds. The extracted reads were used for a new de novo assembly.

### 2.3. Colony Classification and Sample Preparation

**Figure 3:**
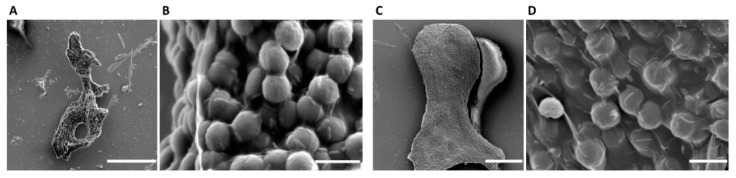
The SEM micrographs of the single *Microcystis* colonies. (**A**) Global view of a *M. aeruginosa* colony. (**B**) Local view of the selected *M. aeruginosa* colony. Bacilliform bacteria were seen on the surface of the polysaccharide shell. (**C**) Global view of a *M. panniformis* colony. (**D**) Local view of the selected *M. panniformis* colony. Bacilliform bacteria were also seen on the surface of the polysaccharide shell. Scale bars in A and C, 100 µm. Scale bars in B and D, 5 µm.

Colonies of *Microcystis* were classified and selected under light microscope and further imaged under scanning electron microscope (SEM) (Figure 3 and Figure S1). *Microcystis* frequently forms large colonies with mucilage and heterotrophic bacteria. Colonies of different species have specific morphological characteristics. The colonies of *M. aeruginosa* form in irregular shape, and the mucilage is frequently broken or perforated, making the population into a network of branches or panes (Figure 3A and Figure S1A). In *M. aeruginosa* colonies, numerous bacteria, which are usually rods, were observed on the surface or embedded in the polysaccharide shell under SEM (Figure 3B). The mucilage of *M. wesenbergii* has clear boundary and is transparent. There are fewer *microcystis* cells of in a *M. wesenbergii* colony, which are sparsely, arranged randomly and formed in hollow population (Figure S1B). Due to the abundance of mucilage and the hollow population, dehydration in the sample preparation of SEM changed the morphology of *M. wesenbergii* colonies, preventing them from being identified under SEM. The colonies of *M. panniformis* are usually flat, with irregular edges and cells arranged densely (Figure 3C and Figure S1C). In SEM micrographs of *M. panniformis* colonies, numerous rod-like bacteria were also observed on the surface or embedded in the polysaccharide shell (Figure 3D).

Colonies of different species were classified under morphological characteristics. Under SEM, plenty of adherent bacteria were observed obviously on the surface of polysaccharide shell of these natural colonies, which revealed that the mutualistic system of *Microcystis* colonies in natural environment were composed of algal cells and abundant heterotrophic bacteria.

### 2.4. Mapping Results

After sequencing, 45 to 166 million reads were generated for each colony (Table 1). Six metagenomes were aligned to NIES843 [3] and FACHB-1757 [29] separately. The mapping results are shown in Table 1. At least 67.87% of the two genomes were covered at least five folds using 45 million reads (Table 1). The high coverage ratio of all metagenomes using both two references revealed that the microbial communities of these metagenomes were mainly composed of *Microcystis* cells. At same coverage, higher coverage ratios were got for each metagenome using FACHB-1757 as reference. The two metagenomes of *M. panniformis* colony both covered over 95% of the FACHB-1757 genome for at least five folds. As a contrast, none of the metagenomes covered over 85% of the NIE-843 genome at same coverage. Similar coverage ratios within species revealed a well consistency between duplicates.

### 2.5. Clustering Results

**Figure 4:**
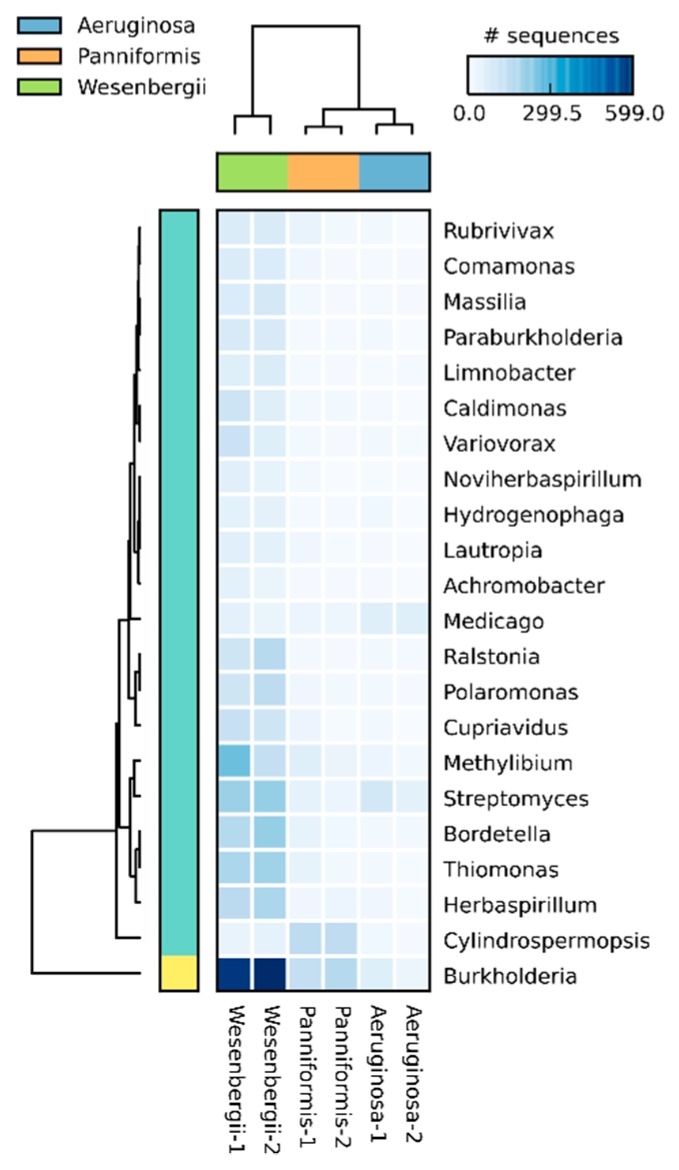
Clustering result of taxonomic analysis results at genus level. Based on the taxonomic analysis results, six samples were clustered at genus level. Metagenomes of the same species showed to be closest with each other. Relatively, the two metagenomes of *M. panniformis* are closer to *M. aeruginosa* than that of *M. wesenbergii*.

After aligning to non-redundant protein database, the relative abundance of orders belonging to Cyanobacteriaidetes were calculated and shown in Figure S3. Taxonomic analysis was performed subsequently using the alignment files. Genera with differential abundance were extracted for clustering analysis (Figure 4). Obviously, metagenomes of the same species show a significantly high similarity. The lineage of each species is distinct from the lineage of the other two species. Relatively, the two metagenomes of *M. panniformis* are closer to *M. aeruginosa* than that of *M. wesenbergii*. The clustering results revealed that single colonies of the same *microcystis* species are similar in the microbial community composition. The relatively close distance between single colonies of *M. panniformis* and *M. aeruginosa* is in accord with their similarity in morphological characteristics.

### 2.6. Assembly and Reads Binning

After quality control and reads trimming, 84 Gbp paired-end sequencing data were used for de novo assembly (Table S1). A total of 105 Mbp of scaffolds with N50 for 4935 bp were assembled using SPAdes. The assembled scaffolds captured 49.83% of all sequenced reads.

To evaluate to bias induced by MDA, a bulk sample was obtained and 6.17 Gbp of paired-end sequencing data were produced. The metagenome of the bulk sample was assembled under the same condition. We calculated the percentage of scaffold with specific GC content in both amplified metagenome and bulk metagenome (Table S2). The results proved that MDA prefers to amplify fragments with low GC content rather than with high GC content. Therefore, we calculated standardization factors for each GC content to normalize the relative abundance.

The binning was based on the different coverage of scaffolds in the data set of each species. Due to the high similarity in microbial composition, only the first metagenomes of each species were used for binning. In the 3D space of coverage, scaffolds clustered together represent putative population genome bins. Further refinement was facilitated for the bins which contained multiple species. In total, eight population bins were identified with GC content ranging from 42.03% to 68.52% and represented with bacterial diversity (five phyla) (Table S3). Six of the population bins were assembled into high-quality genomes (Table 2) which included many rare members with the lowest being 0.126% in relative abundance. In comparison, binning based on GC content and overall coverage which is classically used in metagenomics identified five population bins, and these bins contained more duplicated essential genes which represented multiple species (Figure S4, Table S4). Population bins with similar GC content and overall coverage cannot be separated using classical pipeline. For example, group E in Supplementary Figure 4 which contained 80 duplicated essential genes is the mixture of group 4 (67.60%, GC content) and group 6 (68.52%, GC content). At the phylum level, four of the eight groups were proteobacteria, two were cyanobacteria, one was bacteroidetes, one was gemmatimonadetes and one was the group of euglenozoa. Proteobacteria was discovered to be the most abundant phylum in cyanobacterial water bloom samples, while bacteroidetes was another important phylum [30]. Proteobacteria was also reported to be the most abundant phylum in Lake Taihu [14]. Because of some putative bins, group 3, group 7, and group 8 do not stand out in Figure 1, we highlighted the selected scaffolds of each group in separated figures (Figure S5).

Except for group 3, the genome completeness of the other five groups were estimated to be over 90% based on the number of essential genes, and four of them were over 95% completed (Table 2). The completeness validated using CheckM was in accordance with the results based on essential genes (Table S5). These five genomes have few duplicated essential genes, suggesting that each group had little contamination. However, the contamination validated by CheckM was relatively higher and diverse among genomes (Table S5). The inconsistency in contamination validation might due to the different evaluation criterions used. Different methods had been reported to generate different contamination level [31]. All these results indicated that five pure and nearly completed genomes were obtained through the differential-coverage binning of single *Microcystis* colonies. Group 3 was the most abundant group among the eight discovered groups, and ought to be the most abundant group in all populations in the metagenome according to its quite high relative abundance (15.34%).

At the genus level, group 3 was from *Microcystis*. A lot number of duplicated essential genes of group 3 indicated the genome was not well separated from one the others. During assembly, metagenomes of single colonies of *M. aeruginosa*, *M. wesenbergii* and *M. panniformis* were used unitedly. Group 3 is part of the chimeric genome of *M. aeruginosa*, *M. wesenbergii* and *M. panniformis*.

Group 1 was from *Chryseolinea*, a genus of *Cytophagia*. The GC content of the assembled genome (50.2%) is close to the reported species (49.9%) of *Chryseolinea* which was isolated from soil [32]. Bacteria belonging to *Cytophagia* have been reported to be isolated from *Microcystis* colonies [33,34]. Members of another genus in *Cytophagia*, *Cytophage* act as cyanbacteriolytic bacteria [35], which are able to lyse the cyanobacteria as a food resource. Therefore, the high relative abundance of group 1 in *M. wesenbergii* colony compared with the other two species might be a reason of the sparseness structure. Group 2 was from *Pseudanabaena*, another genus of *Cyanobacteria*. As autotrophic organism, *Pseudanabaena mucicola* can often be found associated to *Microcystis* colonies [36,37,38]. The co-cultivation of *Pseudanabaena* with *Microcystis* strains in the lab revealed strong interaction between these two cyanobacteria, and evidenced that the growth of *Pseudanabaena* in natural systems may cause either *Microcystis* lysis, or colony sedimentation [39]. Group 4 was from *Brevundimonas*, a genus of *Alphaproteobacteria*. *Brevundimonas* has been reported to be a frequenter of *Microcystis* colony associated bacterial in different locations [40]. Group 5 and 6 were from two genera of the same family (*Burkholderiaceae*) in *Betaproteobacteria, Cupriavidus* and *Burkholderia*. Two bacteria in family *Burkholderiaceae* were binned from the metagenome of a lab cultured *Microcystis* stain, *M. wesenbergii* T100 [26]. *Burkholderiales* have been evidenced to be one of the dominated orders in buoyant cyanobacteria [41]. Group 7 was from *Rosemonas*, another genus of *Alphaproteobacteria,* and was also demonstrated to be one of the dominant genera during the algal bloom [42]. Group 8 from *Gemmatimonas* was the lowest abundant population among the eight assembled genomes. *Gemmatimonas* is frequently associated with cyanobacterial colonies [43,44].

## 3. Discussion

*Microcystis* colonies are basic units of cyanobacterial blooms. The colonies are constituted of *Microcystis* cells and heterotrophic bacteria, which vary in size and shape [5]. Morphological characteristics of cells and colonies are considered as species definition of *Microcystis*. The mass *Microcystis* bloom sample is a colony mixture with high complexity. Single colonies in nature are less complex in bacterial composition and more suitable for metagenomic study to reveal the natural mutualistic microsystem. Morphological studies in advance confirmed that colonies were consisted of *Microcystis* cells and numerous heterotrophic bacteria (Figure 3 and Figure S1). After which we constructed a comprehensive pipeline to construct and analyze the metagenomes of these natural mutualistic microsystem.

Due to the trace amount of nucleic acid within a single colony, extracting DNAs step by step is not practicable. Most nucleic acids of the single colony might be lost during extraction and external contaminants might be brought in. Therefore, we lysed single colonies and digested proteins and RNAs in tube. Nucleic acids were amplified using Φ29 DNA polymerase and random hexamer primers. This isothermal amplification has been extensively applied due to the high accuracy and great efficiency [28]. Metagenomes obtained in our study demonstrated that DNAs within single colonies were well amplified using MDA. However, amplification biases among DNA fragments and in GC content have been reported to be induced by MDA [45,46]. To promote the amplification uniformity and to recover more populations within single colonies, improved amplification approach such as micro-channel MDA (µcMDA) [47,48] or droplet MDA are desired [49,50].

Mapping results revealed that the nucleic acids in single *Microcystis* colonies were well recovered by our experimental pipeline. The six metagenomes mapped at least 67.87% of the two reference genomes. Higher coverage of all metagenomes using FACHB-1757 as reference indicated that geographical location is one of the dominant factors in genome similarity. FACHB-1757 is a stain of *M. panniformis* isolated from Lake Taihu in August 2011 [29]. The two metagenomes of *M. panniformis* colony both covered over 95% of the FACHB-1757 genome for at least five folds, indicating that colonies of the same *Microcystis* species at the same geographical location have the highest genome similarity. Metagenomes of the same species have similar coverage ratio regardless of which reference genome was used. Taxonomic analysis of metagenomes after alignment revealed similar results. All these results supported that colonies of the same species are not only consistent in genome, but also similar in microbial community composition.

Metagenomes are complex genome mixtures of individuals. The assembly of metagenomes will result in a large set of independent contigs that are not easily aggregated into individuals [51]. The process which is referred as binning is applied to reduce the complexity of metagenomics data by grouping similar assembled contigs together and annotated to individual genomes [9,12]. Genomic features, such as GC content and tetranucleotide frequency are commonly used in assembly [52]. However, only a few organisms with distinct or even extreme features in base composition could be grouped in complex communities. Herein, we applied an alternative approach to separate organisms by using abundance differences of contigs among samples. This approach is referred as differential-coverage binning, which had been applied in recover genomes from samples processed using two different DNA extraction methods [53] and time series community samples [54]. Colony samples of different *Microcystis* species are the exactly parallel samples for differential-coverage binning. Therefore, we separated population bins by using the differential-coverage of contigs among colonies. In the 3D space of coverage, scaffolds clustered together represent putative population genome bins (Figure 1 and Figure S3). Although scaffolds could be clustered in the 2D space of coverage (Figure S6), 3D space provides a superior binning, in which scaffolds within a putative bin could be screened in the third dimension. Because scaffolds from different microorganisms in one putative bin seriously influence the following assembly result, a purified putative bin means a higher quality genome. The six high-quality genomes assembled demonstrated the high efficiency of our pipeline.

In total, eight population bins were identified which are greatly varied in GC content and abundance, and six of which were assembled into high-quality genomes. All these organisms are the component of the mutualistic system within single *Microcystis* colonies. The constitution of bacteria at the phylum level is consistent with the previous study [14].

## 4. Material and Methods

### 4.1. Sample Collection, Colony Classification, and Observation

A cyanobacterial bloom sample was collected from the water surface using a plastic bucket in Meiliang Bay of Lake Taihu in August 2015, and transported to the laboratory in 50 mL centrifuge tubes. Assemblages were rinsed with 0.02 M phosphate-buffered saline (PBS) to remove free-living bacteria. A bulk sample was obtained to evaluate and standardize the bias of MDA. Centrifuge tubes, culture dishes, and other materials were sterilized and dried before use. Single *Microcystis* colonies were classified based on species features under an optical microscope. *M. aeruginosa* and *M. wesenbergii* colonies were identified referring to Yu et al. [55]. The single colonies which had flattened irregular monolayers with small holes (in old colonies) were identified as *M. panniformis* [29] (Figure S1).

Colonies for SEM observation were prepared as follows. Colonies sample in 1 mL lake water were selected and washed with 0.02 M PBS, and them fixed with 2.5% glutaraldehyde at room temperature for 10 h, and post-fixed in 1% osmium tetroxide at 4 °C for 30 min, followed by rinsing with 0.02 M PBS. After that, the samples were dehydrated by ethanol series (50%, 70%, 95%, and 100%), and dried. Finally, the samples were coated with gold for analysis. The prepared samples were examined under an SEM (S-3000N, Hitachi, Japan).

### 4.2. Colony Lysis and DNA Amplification

The selected single *Microcystis* colonies were rinsed with 0.02 M PBS for three times and with 100 µL TES (50 mM Tris, 1 mM EDTA, 100 mM NaCl) for one time. After removing the supernatant, 5 µL TES and 5 µL lysozyme (10 mg/mL, cat. 62970, Sigma, St. Louis, MO, USA) was added to each colony and the tubes were incubated at 37 °C for 10 h. Subsequently, 0.2 µL proteinase K solution (10 mg/mL, cat. P6556, Sigma) was added to each colony to digest proteins by incubating at 50 °C for 3 h and was inactivated at 95 °C for 10 min. After that, RNAase A (cat. 19101, Qiagen, Hilden, Germany) for 0.1 µL was added to each tube to digest RNAs by incubating at 37 °C for 30 min and was inactivated at 95 °C for 10 min. DNA of the bulk cyanobacterial bloom sample were extracted using E.Z.N.A.^®^ Water DNA Kit (cat. D5525, OMEGA, Norcross, GA, USA).

The 90 µL MDA reaction buffer was added to each lysed colony with a final concentration of 1 × Φ29 buffer (cat. M0269, NEB, Ipswich, MA, USA), 50 µM N6 primer with two phosphorothioate bonds at the 3′ end (Genscript, Nanjing, China), 1 mM dNTP (cat. N0447, Ipswich, MA, NEB) and 1 mg/mL BSA (cat. B9001, Ipswich, MA, NEB). To anneal the random hexamers to single colony DNA, we heated the reaction system at 95 °C for 10 min, and immediately put on ice for at least 30 min. Then 20 units of Φ29 DNA polymerase (cat. M0269, Ipswich, MA, NEB) were added to each tube with brief centrifuging. MDA reactions were carried out at 30 °C for 10 h and terminated at 65 °C for 10 min.

### 4.3. Library Construction and Sequencing

Barcoded paired-end sequencing libraries for 500–700 bp in length were constructed with 2 µg amplified DNA for each colony, and 2 µg DNA for bulk sample according to the manufacturer’s protocol (Illumina, San Diego, CA, USA). The resulting libraries were size-checked by Agilent 2100 Bioanalyzer system (Agilent, Santa Clara, CA, USA). The libraries were then subjected to paired-end sequencing on Illumina X10 platform (Illumina, San Diego, CA, USA) with the 150 bp read option, according to manufacturer’s instructions.

Raw sequence data of the six samples reported in this study have been submitted to the National Center for Biotechnology Information (NCBI) Sequence Read Archive under accessions SRX4337635, SRX4337636, SRX4337637, SRX4337638, SRX4337639, and SRX4337640.

### 4.4. Read Trimming, Alignment

The bioinformatics analysis strategy used to obtain genomes of mutualistic bacteria in single *Microcystis* colonies is detailed in Figure 2. Sequencing reads in FASTQ format were trimmed with trimmomatic-0.36 [56] under following steps and parameters: removing sequencing adapters, cutting the start or end of a read if the quality score is below 3, scanning the reads with a 4-base wide sliding window and cutting the windows whose average quality score per base are below 20, and dropping the reads whose length are below 50 bp.

The trimmed reads were aligned to NIES843 [3] and FACHB-1757 [29] using bwa men [57]. The ratio of aligned reads and coverage of reference were calculated for each metagenome.

### 4.5. Taxonomic Analysis and Clustering

The trimmed reads of each metagenome were aligned to non-redundant protein database (nr) [58] using DIAMOND-0.7.11 [59]. Alignment files were loaded into MEGAN5 [60] to perform taxonomic analysis, and taxonomic information at genus level was extracted. Six metagenomes were aligned separately in this step and clustered by STAMP-2.1.3 [61].

### 4.6. De Novo Metagenome Assembly and Scaffold Coverage

The filtered reads were assembled using SPADes-3.9.0 [62], with a k-mer of 101 and a minimum scaffold length of 1 kbp. The six metagenomes were assembled unitedly.

Reads were mapped to scaffolds using bwa mem [57] and the scaffold coverage information was reported by qualimap-2.1.1 (http://qualimap.bioinfo.cipf.es/). Relative metagenome abundance of each genome bin was calculated as the percentage of metagenome reads mapping to the individual genomes compared to the total number of metagenome reads.

The number of scaffold of specific GC content was counted for both amplified metagenome and bulk metagenome to calculate standardization factor of relative abundance.

### 4.7. Conserved Marker Genes Identification

Firstly, open reading frames were predicted in the assembled scaffolds using the metagenome version of Prodigal [63]. Secondly, proteins were identified using HMMER3 (http://hmmer.janelia.org/) with the default settings by searching a set of 107 HMMs of essential single-copy genes [64] against the predicted open reading frames, except the trusted cutoff was used (-cut_tc). Using BLASTP, the identified proteins were taxonomic classified against the RefSeq (version 84) protein database with a maximum e-value cutoff of 1e-5. Lastly, from the BLAST.xml output file, class level taxonomic assignments were extracted using MEGAN5 [60].

### 4.8. Sequence Binning

Sequence binning was facilitated by using the metagenomes generated from single *Microcystis* colonies of three species. The first metagenomes of the two duplicates of each species were selected for differential-coverage binning. Difference in colony-specific bacteria abundance among the three single *Microcystis* colonies resulted in different relative abundance of the populations in the three metagenomes. By independently mapping the reads from each selected metagenome to the assembled scaffolds, coverages of scaffolds in the three metagenomes were calculated. Distinct grouping was identified by plotting the three coverage against one the others in R. The grouped scaffolds suggest that they own similar coverage in all three metagenomes, indicating that they are from the same species. Initial groups were selected by coloring scaffolds according to their taxonomic affiliation as determined above. For some groups which contain a lot of duplicated genes, tetranucleotide frequencies and GC content were used in the next step to purify these initial groups.

For comparison, scaffolds were also binned based on their GC content and overall coverage.

### 4.9. Re-Assembly

Re-assembly was performed by extracting reads mapping to scaffolds of a particular bin and all associated paired-end reads. The extracted reads were used for a new de novo assembly using SPADes-3.9.0 [62] with a k-mer of 101. The expected coverage and coverage cutoff parameters were adjusted individually by running multiple assemblies. To validate the assemblies, CheckM was used to assess genome quality [65].

## 5. Conclusions

In this work, we proposed a universal metagenome construction and analysis pipeline for uncultured single *Microcystis* colonies, the basic mutualistic units in cyanobacterial blooms. By the isothermal DNA amplification and differential-coverage binning of scaffolds, six high-quality genomes (completeness > 90%) were assembled, including some rare members (< 1%). The analysis of wild single *Microcystis* colonies improved our understanding of the mutualistic relationship within *Microcystis* colonies and the mechanism of cyanobacterial blooms.

## Figures and Tables

**Table 1:** Data summary and mapping results.

	Aeruginosa-1	Aeruginosa-2	Wesenbergii-1	Wesenbergii-2	Panniformis-1	Panniformis-2
Total reads (million)	94.68	77.94	45.23	165.66	106.81	91.77
Coverage Ratio of NIES-843*	67.87%	69.71%	83.83%	78.41%	80.56%	75.70%
Coverage Ratio of FACHB-1757 *	68.20%	70.35%	87.09%	94.30%	99.89%	95.32%

* With 45 million reads in 5× coverage.

**Table 2:** Assembly statistics for the 6 representative genomes.

Figure ID	No. Contigs	Total Length (bp)	GC (%)	No. Essential Genes	No. Duplicated Essential Genes	Standardized Relative Abundance* (%)				Genus	Phylogenetic Affiliation
						*aeruginosa*	*wesenbergii*	*panniformis*	Overall		
1	149	3,487,294	50.16	101/105	1	0.004	2.250	0.116	1.004	*Chryseolinea*	Bacteroidetes
2	309	4,301,792	42.03	102/106	5	0.190	0.528	0.095	0.443	*Pseudanabaena*	Cyanobacteriaidetes
3	3434	10,835,247	43.20	81/106	40	9.356	23.222	8.004	15.344	*Microcystis*	Cyanobacteroidetes
4	160	2,863,377	67.60	98/106	5	0.003	0.567	0.064	0.376	*Brevundimonas*	Proteobacteria
5	39	1,954,923	54.00	102/106	1	0.007	0.435	0.004	0.126	*Cupriavidus*	Proteobacteria
6	219	3,678,702	68.52	103/106	2	0.040	0.774	0.094	0.842	*Burkholderia*	Proteobacteria

* Standardized relative abundance was calculated as the standardized percentage of reads of a genome bin in the total number of reads or the number of reads in a specific metagenome.

## Supplementary Materials

Supplementary materials can be found at https://www.mdpi.com/1422-0067/20/20/5047/s1.

## Abbreviations

MDA	multiple displacement amplification
3D	three-dimension
SEM	scanning electron microscope
µcMDA	micro-channel MDA
PBS	phosphate-buffered saline
NCBI	National Center for Biotechnology Information
