# Supplementary material for: Obtaining Genome Sequences of Mutualistic Bacteria in Single Microcystis Colonies
Source: Int J Mol Sci. 2019 Oct 11;20(20):5047. doi: 10.3390/ijms20205047 (PMC6829522; doi:10.3390/ijms20205047)
Supplement: Supplementary file 1 [file ijms-20-05047-s001.pdf]

Supplementary Material

There are six figures and five tables in this supplementary material.

Supplementary Figure S1. Single Microcystis colony under optical microscope.

Supplementary Figure S2. The other view of Sequence 3D coverage binning of metagenome scaffolds from the single Microcystis colonies.

Supplementary Figure S3. The percent abundance of orders belonging to Cyanobacteriaidetes.

Supplementary Figure S4. Binning of metagenome scaffolds based on GC content and overall coverage.

Supplementary Figure S5. Selected scaffolds of three putative population bins.

Supplementary Figure S6. Coverage binning of metagenome scaffolds from the single Microcystis colonies.

Supplementary Table S1. *De novo* assembly statistics of the metagenome.

Supplementary Table S2. Scaffold distribution in GC content

Supplementary Table S3. Assembly statistics for all 8 genomes.

Supplementary Table S4. Assembly statistics based on GC content and overall coverage.

Supplementary Table S5. Completeness and strain heterogeneity of putative genomes

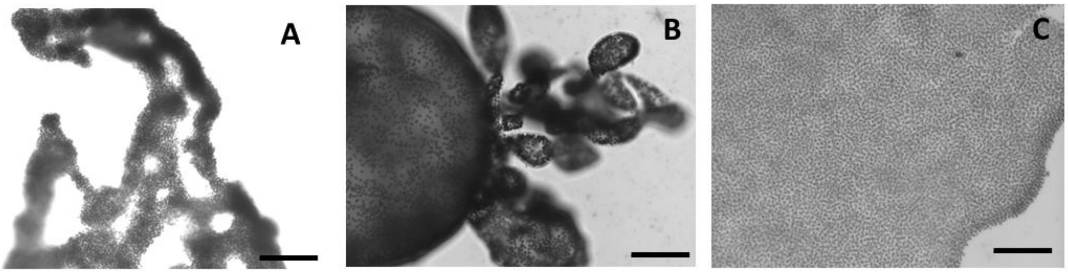

Supplementary Figure S1. Single Microcystis colonies under optical microscope. A. M. aeruginosa colony. B. *M. wesenbergii* colony. C. *M. panniformis* colony. (scale bar, 100 μm)

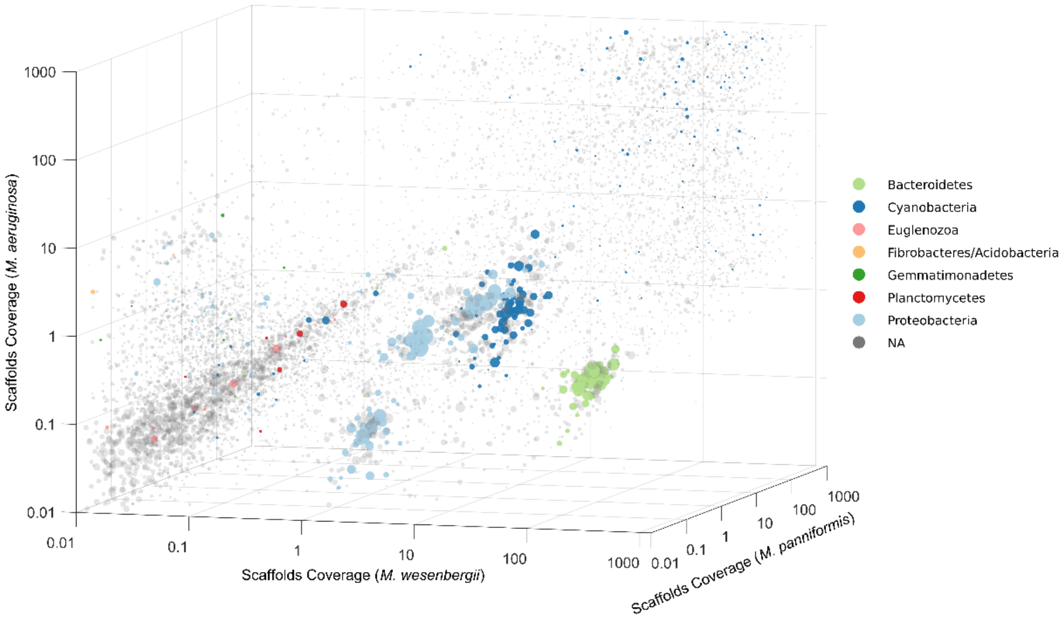

Supplementary Figure S2. The other view of sequence 3D coverage binning of metagenome scaffolds from the single Microcystis colonies. Plots represent scaffolds, the size indicates the length of scaffolds and the color shows the phylum-level taxonomic affiliation. The gray spots are scaffolds which cannot assign to the essential genes of the 8 most abundant phyla. Clusters of similarly colored spots represent potential genome bins. Spots in similar coverage pattern, such as around diagonal, were extracted and further binned based on the differences in nucleotide composition.

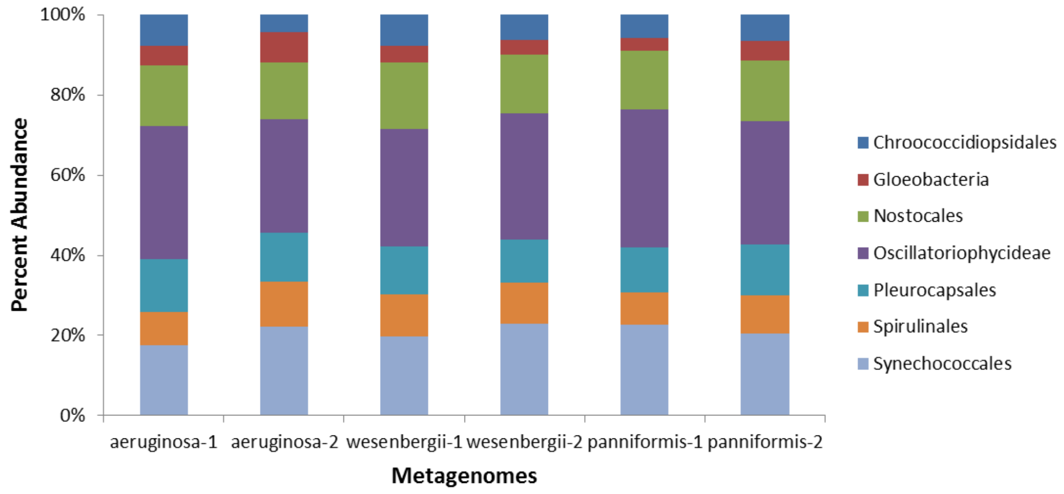

Supplementary Figure S3. The percent abundance of orders belonging to Cyanobacteriaidetes. The abundance of each order was taken logarithm before calculating percentage.

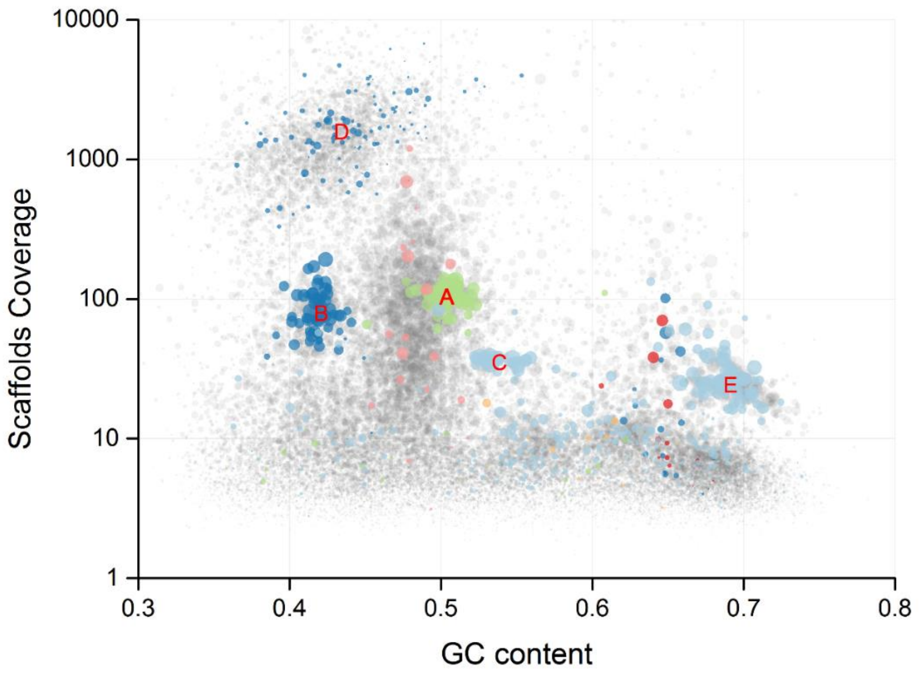

Supplementary Figure S4. Binning of metagenome scaffolds based on GC content and overall coverage.

A

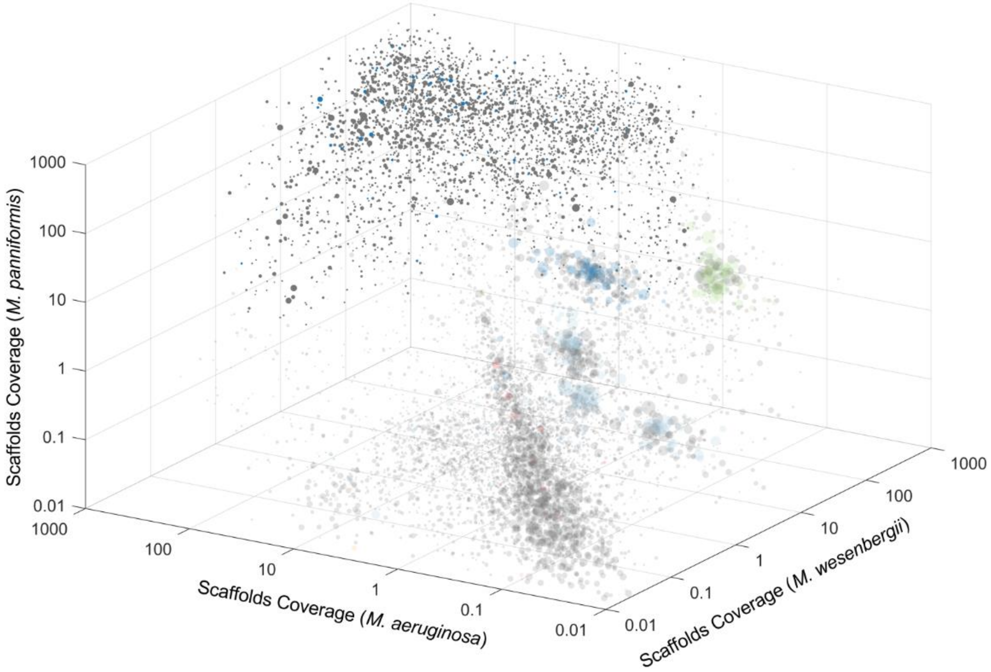

Scaffolds Coverage (M. panniformis)
1000
100
10
1
0.1
0.01
1000
100
10
1
0.1
0.01
Scaffolds Coverage (M. aeruginosa)
0.01
0.1
1
10
100
1000
Scaffolds Coverage (M. wesenbergii)

B

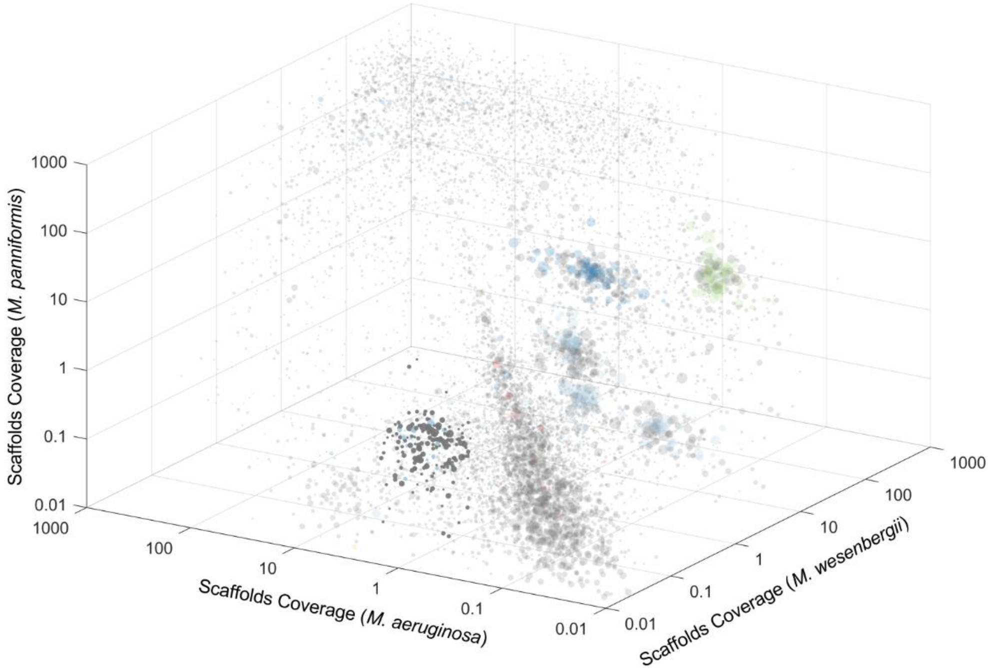

Scaffolds Coverage (M. panniformis)
1000
100
10
1
0.1
0.01
1000
100
10
1
0.1
0.01
Scaffolds Coverage (M. aeruginosa)
0.01
0.1
1
10
100
1000
Scaffolds Coverage (M. wesenbergii)

C

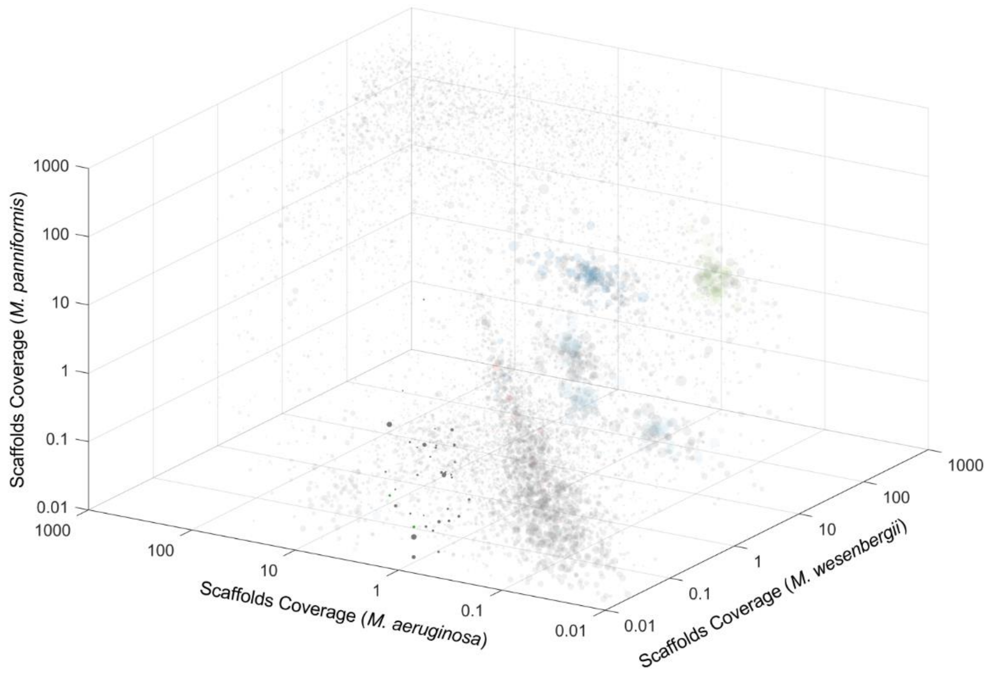

Supplementary Figure S5. Selected scaffolds of three putative population bins. **A.** Selected scaffolds of group 3 (Bold plots). **B.** Selected scaffolds of group 7 (Bold plots). **C.** Selected scaffolds of group 8 (Bold plots).

A

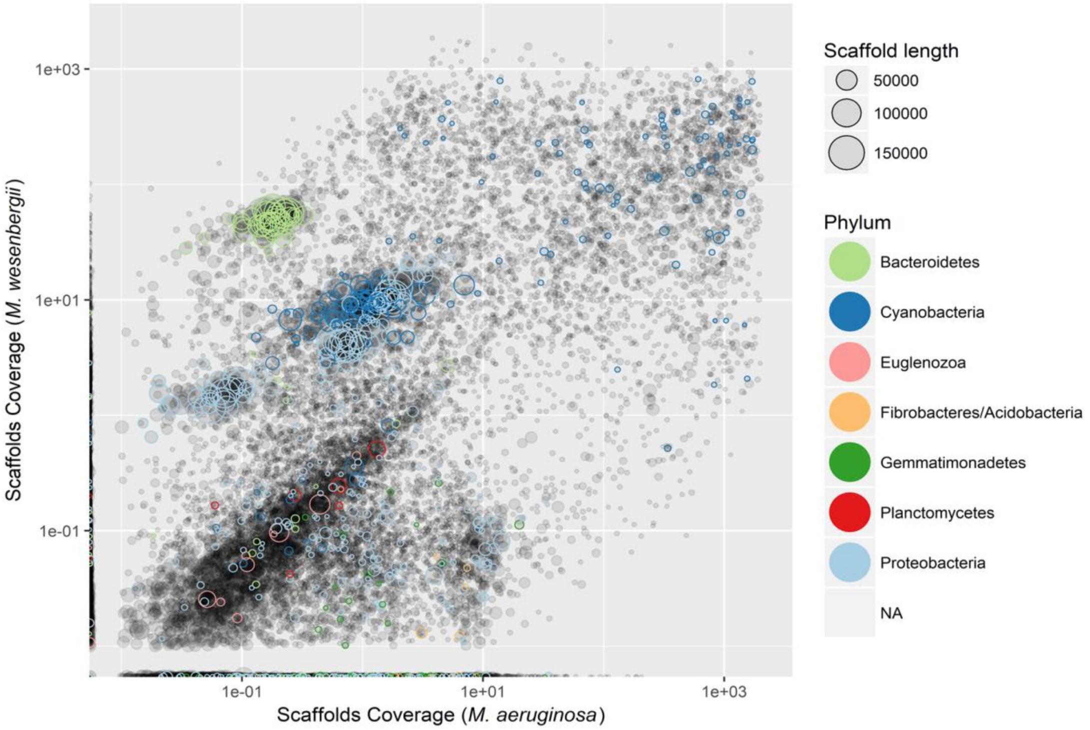
Scaffold length
50000
100000
150000
Phylum
Bacteroidetes
Cyanobacteria
Euglenozoa
Fibrobacteres/Acidobacteria
Gemmatimonadetes
Planctomycetes
Proteobacteria
NA
1e+03
1e+01
1e-01
Scaffolds Coverage (M. wesenbergii)
1e-01
1e+01
1e+03
Scaffolds Coverage (M. aeruginosa)

B

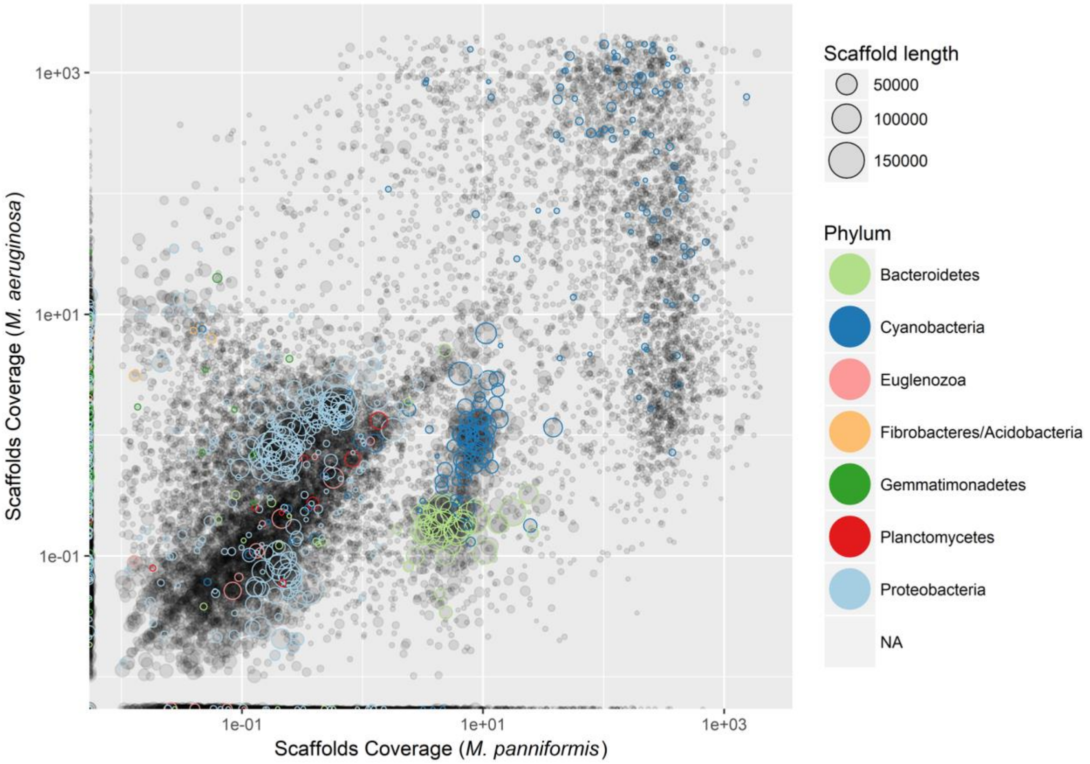
Scaffold length
50000
100000
150000
Phylum
Bacteroidetes
Cyanobacteria
Euglenozoa
Fibrobacteres/Acidobacteria
Gemmatimonadetes
Planctomycetes
Proteobacteria
NA
1e+03
1e+01
1e-01
Scaffolds Coverage (M. aeruginosa)
1e-01
1e+01
1e+03
Scaffolds Coverage (M. panniformis)

C

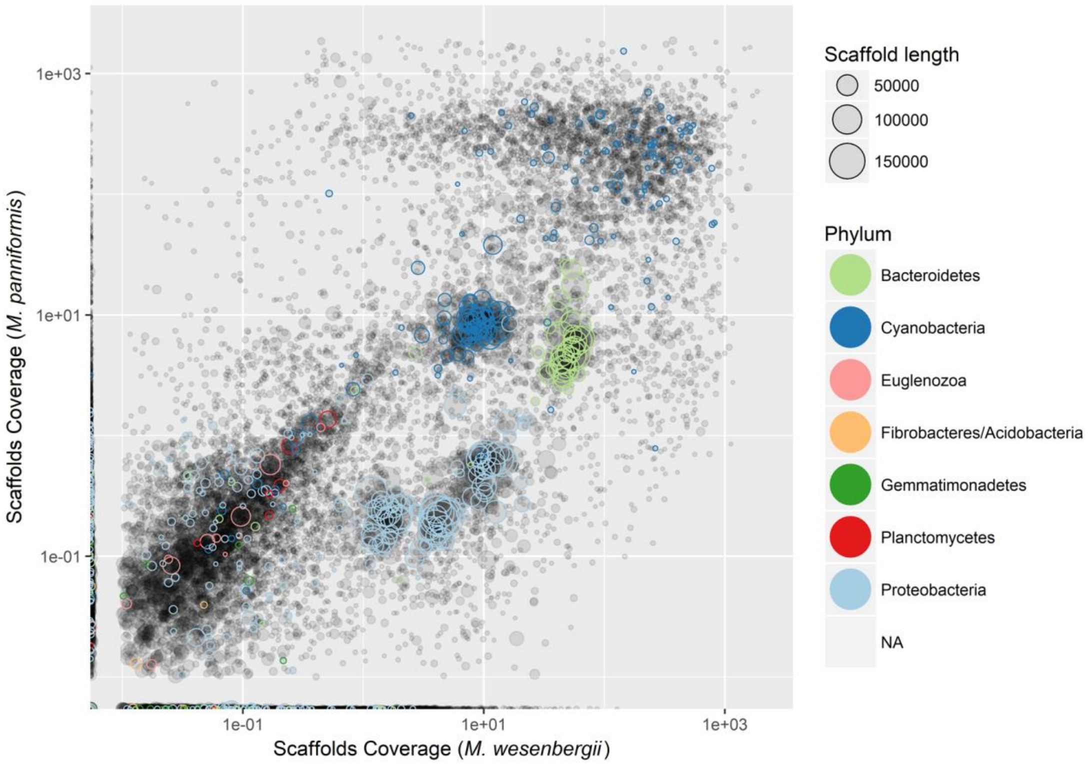

Supplementary Figure S6. Coverage binning of metagenome scaffolds from the single Microcystis colonies. **A.** Scatter plots of coverage between metagenome of *M. aeruginosa* and *M. wesenbergii*. **B.** Scatter plots of coverage between metagenome of *M. panniformis* and *M. wesenbergii*. **C.** Scatter plots of coverage between metagenome of *M. wesenbergii* and *M. panniformis*. Plots represent scaffolds, the size indicates the length of scaffolds and the color shows the phylum-level taxonomic affiliation. The gray spots are scaffolds which cannot assign to the essential genes of the 8 most abundant phyla. Clusters of similarly colored spots represent potential genome bins.

Supplementary Table S1. *De novo* assembly statistics of the metagenome. Quality trimming of the raw data was performed before assembly.

	**The whole metagenome**
Metagenome size (Gbp)	84
Average read length (bp)	144
Total assembly size (Mbp)*	105
N50 (bp)*	4,935
Max scaffold size (bp)	198,017
Scaffold Number*	31,573
Reads used in the assembly	49.83%

*Only scaffolds ≥1 Kbp were considered.

Supplementary Table S2. Scaffold distribution in GC content

GC content		20%-30%	30%-40%	40%-50%	50%-60%	60%-70%	70%-80%
# Scaffold (MDA)		6	2982	12893	6450	7994	1248
		0.02%	9.44%	40.84%	20.43%	25.32%	3.95%
# Scaffold (Bulk)		40	2803	6615	7641	13909	6208
		0.11%	7.53%	17.77%	20.53%	37.37%	16.68%
Standardization Factor		5.5000	0.7977	0.4351	1.0049	1.4759	4.2228

Supplementary Table S3. Assembly statistics for all 8 genomes.

Figure ID	No. contigs	Total length (bp)	GC (%)	No. essential genes	No. duplicated essential genes	Relative abundance* (%)				Genus	Phylogenetic affiliation
						aeruginosa	***wesenbergii***	***panniformis***	**Overall**		
1	149	3,487,294	50.16	101	1	0.004	2.250	0.116	1.004	*Chryseolinea*	Bacteroidetes
2	309	4,301,792	42.03	102	5	0.190	0.528	0.095	0.443	*Pseudanabaena*	Cyanobacteriaidetes
3	3434	10,835,247	43.20	81	40	9.356	23.222	8.004	15.344	*Microcystis*	Cyanobacteroidetes
4	160	2,863,377	67.60	98	5	0.003	0.567	0.064	0.376	*Brevundimonas*	Proteobacteria
5	39	1,954,923	54.00	102	1	0.007	0.435	0.004	0.126	*Cupriavidus*	Proteobacteria
6	219	3,678,702	68.52	103	2	0.040	0.774	0.094	0.842	*Burkholderia*	Proteobacteria
7	211	931,661	61.71	34	3	0.008	0.067	0.004	0.280	*Roseomonas*	Proteobacteria
8	105	322,085	66.81	19	0	0.003	0.017	0.000	0.007	*Gemmatimonas*	Gemmatimonadetes

*Relative abundance was calculated as the percentage of reads of a genome bin in the total number of reads or the number of reads in a specific metagenome.

Supplementary Table S4. Assembly statistics based on GC content and overall coverage.

Figure ID	No. contigs	Total length (bp)	GC (%)	No. essential genes	No. duplicated essential genes	Relative abundance* (%)				Phylogenetic affiliation
						aeruginosa	*wesenbergii*	*panniformis*	Overall	
A	441	6,039,631	50.05	101	5	1.092	2.463	0.120	1.260	Bacteroidetes
B	718	5,897,060	41.90	106	6	0.709	1.685	0.309	2.626	Cyanobacteriaidetes
C	3870	10,366,646	43.40	82	45	25.605	44.249	20.373	37.906	Cyanobacteriaidetes
D	109	2,022,017	53.83	104	5	0.031	0.491	0.005	0.198	Proteobacteria
E	435	7,049,124	68.88	106	80	0.044	1.161	0.133	0.898	Proteobacteria

*Relative abundance was calculated as the percentage of reads of a genome bin in the total number of reads or the number of reads in a specific metagenome.

Supplementary Table S5. Completeness and strain heterogeneity of putative genomes

Figure ID	Phylogenetic affiliation	Completeness	Contamination	Strain heterogeneity
1	Bacteroidetes	100.00%	47.20%	16.33
2	Cyanobacteroidetes	96.55%	50.84%	34.21
3	Cyanobacteroidetes	99.84%	148.67%	86.54
4	Proteobacteria	93.10%	121.23%	21.44
5	Proteobacteria	94.83%	50.72%	36.30
6	Proteobacteria	98.28%	112.46%	15.60
7	Proteobacteria	30.96%	27.06%	26.14
8	Gemmatimonadetes	17.16%	9.97%	46.34
